# Markers of Subclinical Atherosclerosis in Severe Obesity and One Year after Bariatric Surgery

**DOI:** 10.3390/jcm11082237

**Published:** 2022-04-16

**Authors:** Nina Kovac, Lisa M. D. Grymyr, Eva Gerdts, Saied Nadirpour, Bjørn G. Nedrebø, Johannes J. Hjertaas, Knut Matre, Dana Cramariuc

**Affiliations:** 1 Department of Clinical Science, University of Bergen, 5021 Bergen, Norway; nina.kovac@student.uib.no (N.K.); lisa.grymyr@uib.no (L.M.D.G.); eva.gerdts@uib.no (E.G.); bjorn.gunnar.nedrebo@helse-fonna.no (B.G.N.); knut.matre@uib.no (K.M.); 2 Department of Heart Disease, Haukeland University Hospital, 5021 Bergen, Norway; 3 Department of Medicine, Haugesund Hospital, 5528 Haugesund, Norway; saied.nadirpour@helse-fonna.no (S.N.); johannes.hjertaas@gmail.com (J.J.H.)

**Keywords:** obesity, atherosclerosis, valve sclerosis, pericardial fat, aortic wall

## Abstract

Background: Aortic valve sclerosis (AVS), mitral valve sclerosis (MVS), remodeling of major arteries, and increased pericardial fat are associated with subclinical atherosclerosis. We assessed these markers of atherosclerosis in severely obese patients before and 1 year after bariatric surgery. Methods: Eighty-seven severely obese patients (43 ± 10 years, preoperative body mass index [BMI] 41.8 ± 5 kg/m^2^) underwent echocardiography before and 1 year after Roux-en-Y bypass surgery in the FatWest (Bariatric Surgery on the West Coast of Norway) study. We measured the end-diastolic aortic wall thickness (AWT), pericardial fat thickness at the right ventricular free wall, and AVS/MVS based on combined aortic leaflet thickness and hyperechoic valve lesions. Results: Postoperatively, patients experienced a reduction of 12.9 ± 3.9 kg/m^2^ in BMI, 0.5 ± 1.9 mm in AWT, 2.6 ± 2.3 mm in pericardial fat, and 45%/53% in AVS/MVS (*p* < 0.05). In multivariate regression analyses with adjustment for clinical and hemodynamic variables, less pericardial fat reduction was associated with male sex and higher 1-year blood pressure and BMI, and less AWT-reduction with higher age and 1-year BMI (*p* < 0.05). Persistent AVS and MVS were related to higher 1-year BMI and more advanced valve sclerosis preoperatively (*p* < 0.05). Conclusions: Markers of subclinical atherosclerosis decreases significantly 1 year after bariatric surgery, particularly in younger patients that achieve a BMI < 28 kg/m^2^.

## 1. Introduction

Obesity and the associated inflammation accelerate atherosclerosis in all its phases, from early fatty streak development to atherothrombosis [1,2]. Obese patients often exhibit clustering of metabolic cardiovascular (CV) risk factors that partially mediate this relation as hypertension, dyslipidemia and hyperglycemia. Yet obesity also carries an independent atherosclerotic risk linked mainly to the extent and metabolic activity of visceral adipose tissue [3]. In particular, the association between visceral adipose tissue and an increased risk of coronary artery disease (CAD) has been studied extensively during the past few years.

Identification of subclinical atherosclerosis by imaging modalities significantly improves the prediction of CAD risk compared to the assessment of clinical risk factors alone [4,5]. Quantification of coronary artery calcification by computed tomography (CT) is the most commonly used modality to assess subclinical disease [5], but its use is restricted by cost, availability, and radiation exposure. Detection of aortic (AVS) and mitral valve sclerosis (MVS) by echocardiography, defined from leaflet thickening and calcification, as well as from changes in major arteries’ wall structures, has strong CV risk predictive value independent of traditional risk factors and CT coronary artery calcification [4], and is particularly useful when CT is not available.

Obesity increases the risk of AVS in the general population [6] and causes a thicker aortic wall in experimental studies [7]. It also involves higher pericardial fat mass, a marker of visceral adiposity, increased coronary plaque burden [8,9,10], and higher risk of acute coronary syndromes and heart failure [11,12]. Although data from small-scaled studies show that weight reduction through lifestyle intervention or surgery [13,14] reduces the pericardial fat mass, previous studies have not addressed the reversibility of valve sclerosis and aortic root remodeling after large weight loss following bariatric surgery.

Mortality due to CAD has declined over the past few decades due to better in-hospital patient management, yet the ongoing global obesity epidemic and population ageing are expected to result in a rising number of CAD patients requiring hospitalization, revascularization procedures and drug treatment [15]. Thus, quantification of subclinical atherosclerosis in obese subjects and after weight-reducing interventions is clinically and epidemiologically important. In the present study, we analyzed the 1-year impact of bariatric surgery on the extent of subclinical atherosclerosis estimated by echocardiography from AVS, MVS, aortic root wall thickness (AWT), and pericardial fat in 87 patients with initially severe obesity.

## 2. Materials and Methods

### 2.1. Study Population

The FatWest (Bariatric Surgery on the West Coast of Norway) study recruited prospectively patients with severe obesity living on the West Coast of Norway and referred for a Roux-en-Y gastric bypass [16]. Of these patients with a standard indication for bariatric surgery according to national guidelines [17], 123 were examined clinically by echocardiography and blood samples at study baseline (2–3 months before surgery) and 6 months, 1, 2 and 5 years postoperatively, as previously reported [17,18]. From the baseline cohort, 95 patients presented at the 1-year postoperative visit. In these, echocardiographic assessment of markers of atherosclerosis at 1 year was not possible in eight patients (not measurable aortic wall thickness and/or AVS) resulting in a final population size of 87 patients.

Hypertension was considered present if patients had a history of hypertension, blood pressure (BP) ≥ 140/90 mmHg at the baseline clinical visit, and/or were treated with antihypertensive drugs. Diabetes mellitus was present in patients with high-fasting blood sugar according to the WHO definition, previously diagnosed diabetes, or the use of antidiabetic medications.

The study was conducted in accordance with the revised Declaration of Helsinki. All patients signed a written informed consent, and the study was approved by the regional ethics committee (registration number 2021/22196).

### 2.2. Echocardiographic Protocol

All studies were performed on the same Vivid E9 scanner following a standardized echocardiographic protocol (GE Vingmed Ultrasound AS Horten, Norway) [17,18]. Echocardiographic images were first analyzed by a junior researcher (LMDG), and thereafter quality assured by a single senior researcher (DC) on Image Arena workstations (Image Arena 4.6, Tomtec, Unterschleissheim, Germany) at the study core laboratory in Bergen, Norway.

Measurements of left atrial and left ventricular (LV) dimensions and mass were performed following the joint American Society of Echocardiography and European Association of Cardiovascular Imaging guidelines [19]. LV hypertrophy was defined based on LV mass indexed for height^2.7^ (cutoff: ≥46.7 g/m^2.7^ in women and ≥49.2 g/m^2.7^ in men), as recommended in obese subjects [19,20]. AWT was measured as the anterior root wall thickness at the level at the largest sinus lumen diameter in a zoomed two-dimensional parasternal long-axis view, at end-diastole. AVS and MVS were graded as absent, mild, moderate, or severe, based on the combined assessment of leaflet thickness and hyperechoic valve and/or annular lesions, as previously described [21]. Pericardial fat was measured as the thickness of combined epicardial (between the right ventricular epicardium and the visceral pericardium) and pericardial fat (between the visceral and parietal pericardium) in a parasternal long-axis view at end-diastole. This approach avoids underestimations that might occur when pericardial borders are not clearly visualized [22] and for better agreement with pericardial fat thickness assessed by CT [23] and magnetic resonance imaging [10].

### 2.3. Laboratory Analyses

Lipid profile, serum creatinine, 25-hydroxyvitamin D, and glycated haemoglobin (HbA_1c_) were analyzed in all patients at baseline and at the 1-year postoperative visit.

### 2.4. Statistical Analyses

All analyses were performed using IBM SPSS Statistics 26.0 (IBM, Armonk, NY, USA). Data are presented as mean ± standard deviation (SD) for continuous variables, and as percentages for categorical variables. Clinical and echocardiographic findings at the two study visits were compared using paired samples *t*-tests or chi-square tests. Factors associated with pre- and postoperative AVS, MWS, AWT, and pericardial fat thickness were identified by Spearman correlation tests and univariable regression analyses. Moreover, AVS, MWS, AWT, and pericardial fat thickness at the 1-year postoperative visit were compared between patients with postoperative BMI below and above the median 1-year value. Covariates of change in pericardial fat thickness and AWT were tested in multivariable linear regression analyses with backward stepwise selection of covariates among the following parameters: age; sex; preoperative diabetes, BP, BMI, pericardial fat, and AWT; 1-year BMI, BP and 1-year reduction in heart rate and LV mass. Results are presented as standardized β coefficients with *p* values. Similarly, covariates of persistent AVS and MVS at the 1-year visit were identified in backward stepwise logistic regression analyses among the following: age; sex; preoperative diabetes, BP, BMI and pericardial fat thickness; 1-year BP, BMI and pericardial fat thickness. Results are presented as Wald, odds ratios (ORs) with 95% confidence interval (CI) and *p* values of significance. Covariates in all multiple regression models were selected based both on clinical relevance and a *p* < 0.1 level of significance for the association with the dependent variables in univariate analyses. A two-tailed *p* < 0.05 was considered significant in all analyses.

### 2.5. Patient and Public Involvement

There was no patient or public engagement in the design or conduct of this study.

## 3. Results

### 3.1. Clinical Characteristics

The study population was on average 43 ± 10 years old at baseline, included 70% women, and had a median preoperative BMI of 41.2 kg/m^2^ in both sexes. During the first 14 ± 3 months following Roux-en-Y gastric bypass, the patients experienced a decrease of 37 ± 12 kg in body weight, and 8 ± 10 mmHg and 4 ± 6 mmHg in systolic and diastolic BP, respectively (all *p* < 0.001, Table 1). Their 1-year use of antihypertensive, antidiabetic and lipid-lowering medication was halved compared to baseline, while their metabolic profile and serum 25-hydroxyvitamin D levels improved (Table 1). Women and men had a similar 1-year BMI-reduction: 12.8 vs. 13.0 kg/m2, respectively (*p* = 0.78).

### 3.2. Preoperative Markers of Atherosclerosis

**Figure 1:**
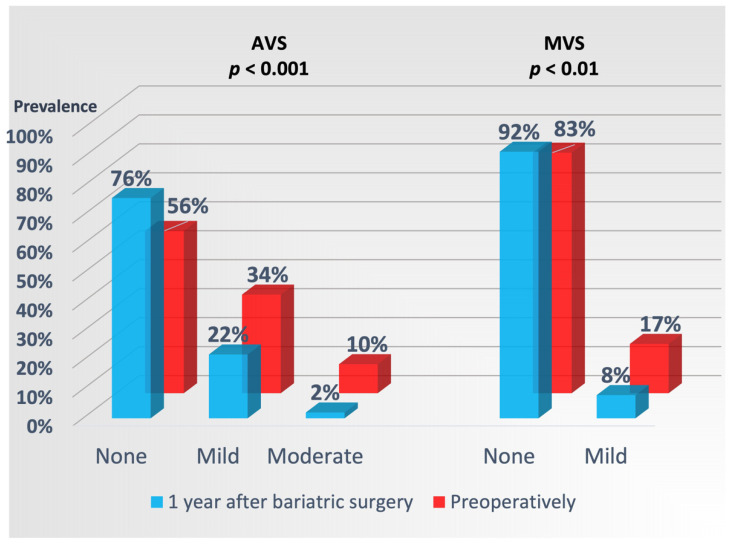
Prevalence of AVS and MVS preoperatively and 1 year after bariatric surgery. *p* values of significance for reduction in AVS and MVS after surgery, respectively.

Left-sided valve sclerosis was present in 56% of the patients preoperatively, with AVS being the most common valvular abnormality (Figure 1), and equally distributed between sexes and patients with vs. without hypertension and diabetes, respectively. Prevalence of AVS increased with higher HbA_1c_ (r = 0.23, *p* = 0.05), but not with higher serum total cholesterol or triglycerides levels. Baseline MVS was less prevalent than AVS and only of a mild degree (Figure 1), but its presence was significantly correlated with higher BMI (r = 0.22, *p* < 0.05).

**Figure 2:**
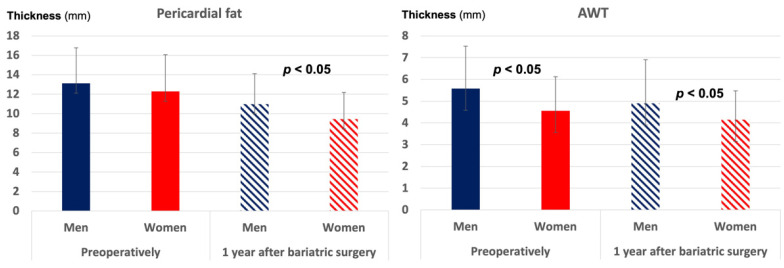
Pericardial fat thickness and AWT (in mm) in women and men preoperatively and 1 year after bariatric surgery. The bars indicate the mean values, and the error bars the standard deviations. *p* values of significance for comparison between men and women at each study visit.

Pericardial fat was abundant in the whole population prior to surgery, with a thicker fat layer being associated with the presence of diabetes (r = 0.23, *p* = 0.03), thicker AWT (r = 0.33, *p* = 0.02) and higher HbA_1c_ (r = 0.29, *p* = 0.02). Men had numerically more pericardial fat than women (Figure 2) and significantly thicker AWT (5.58 mm vs. 4.56 mm, *p* < 0.05, Figure 2).

### 3.3. Markers of Atherosclerosis 1 Year after Bariatric Surgery

**Figure 3:**
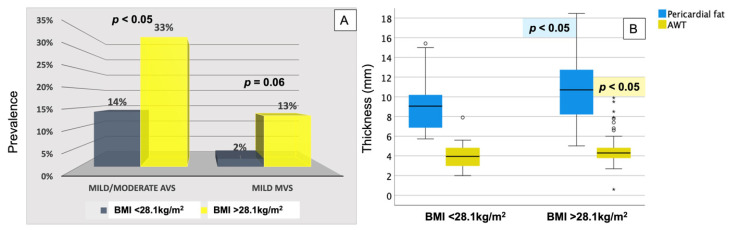
One-year postoperative AVS and MVS (percentages, panel (**A**)), and pericardial fat thickness and AWT (in mm, data presented as boxplots in panel (**B**)) in patients with lower vs. higher than the median achieved BMI of 28.1 kg/m^2^. *p* values of significance for comparison of each marker of atherosclerosis between the two groups.

All echocardiographic markers of subclinical atherosclerosis decreased significantly 1 year after bariatric surgery, with the lowest 1-year prevalence in patients with a postoperative BMI below the median of 28.1 kg/m^2^ (Table 1, Figure 3).

Diabetic and hypertensive patients, as well as those with higher preoperative BMI (> the preoperative median of 41.5 kg/m^2^) experienced less reduction in pericardial fat thickness and AWT. Men retained a significantly thicker pericardial fat layer and AWT than women after surgery (Figure 2), while patients with preoperative BMI > 41.5 kg/m^2^ had less improvement in MVS (*p* < 0.05).

In multivariate regression analyses with adjustment for multiple clinical and hemodynamic variables, reduction in pericardial fat was most pronounced in women and in those with the thickest preoperative fat layer, and negatively associated with higher diastolic BP and higher BMI 1 year after surgery (Table 2). In a similar model, reduction in AWT was negatively associated with higher age and higher BMI 1 year after surgery (Table 2). Additional adjustments for type of medical treatment, HbA_1c_, and lipid profile did not change these results.

In binary logistic regression analyses with stepwise removal of non-significant variables, persistent AVS and MVS 1 year after bariatric surgery were associated with higher 1-year BMI and presence of more advanced valve sclerosis at baseline (Table 3). Additionally, higher age was independently associated with persistent AVS (Table 3).

## 4. Discussion

Subclinical atherosclerosis is associated with left-sided heart valve sclerosis, increased amount of pericardial fat, and remodeling of the ascending aorta at conventional echocardiography [11,23,24]. The presence of these markers of subclinical atherosclerosis signals a high risk of CV events [4]. We demonstrate in the current analysis that patients with severe obesity have a large prevalence of valve sclerosis for age in addition to a large amount of pericardial fat, and thickened aortic root wall, particularly in men and diabetic patients. This is to our best knowledge the first study to show that large weight loss 1 year after bariatric surgery partially reverses these markers of subclinical atherosclerosis, adding a pathophysiological explanation to the previously observed reduced incidence of acute myocardial infarction and coronary revascularization procedures up to 11 years after weight-reducing surgery in retrospective studies [25,26].

### 4.1. Valve Sclerosis in Severe Obesity and after Bariatric Surgery

AVS is common in elderly individuals and typically detected by cardiac imaging performed on other indications due to the asymptomatic nature of valve sclerosis. However, its presence is a predictor of a higher risk of coronary events and death after adjustment for traditional risk factors [27] and thus a marker of subclinical organ damage detectable by standard echocardiography. Obesity of genetic causes has been causally linked to a higher risk of aortic valve stenosis, particularly when BMI exceeds 35 kg/m^2^ and at a higher waist-hip ratio reflecting increased abdominal visceral fat [6]. However, no previous studies have investigated how frequent AVS is among individuals with severe obesity and the reversibility of this finding. In our patient population with a standard indication for bariatric surgery, we found a 44% preoperative prevalence of AVS, corresponding to findings in 20 years older healthy individuals in the Northern Manhattan Study [27], but with a significant fall to 24% prevalence 1 year after surgery, thus reducing the age gap towards the general population with about 8 years [27]. Persistent AVS at the 1-year control was independently related to still higher BMI despite important weight loss and more advanced AVS at study inclusion. While calcifications theoretically are not expected to regress, earlier AVS stages with valve thickening at echocardiography can reflect ongoing metabolic disturbances (reflected among others in higher HbA_1c_) promoting lipid infiltration and valvular inflammation. Both bioactive lipids and inflammatory cells have previously been identified in explanted stenotic aortic valves [28,29]; however, the earlier stages of human AVS have been less available for pathological studies and are thus less explored. Our findings support a potential for resolution of early valvular sclerotic lesions in severely obese subjects.

MVS has a lower prevalence in the general population, but it is strongly associated with higher coronary and CV risk, also after correction for traditional risk factors and the presence of coronary artery calcifications [4]. Even if its association with obesity has been less explored, a higher rate of metabolic syndrome and total body fat has previously been reported in patients with MVS [30]. In our group of severely obese patients, MVS was twice as frequent as to what has been reported in asymptomatic, normal weight individuals [30], but, similarly to AVS, its prevalence halved 1 year after bariatric surgery. Persistent postoperative MVS was found in patients with more severe valve sclerosis preoperatively, and higher postoperative BMI, consistent with findings in the aortic valve.

### 4.2. Pericardial Fat in Severe Obesity and after Bariatric Surgery

Previous studies have well documented that the amount of pericardial fat increases with BMI, and that the epicardial fat layer produces proinflammatory adipokines promoting local inflammation and coronary atherosclerosis [8,9]. In 24 men with mild obesity, 3 months of aerobic training has been shown to reduce the layer of pericardial fat from 8.1 to 7.4 mm on average [13]. A retrospective analysis of 23 patients undergoing bariatric surgery also found that the epicardial fat layer diminished from 5.3 to 3.0 mm 8 months after surgery. By analyzing the total thickness of the pericardial fat layer for improved feasibility [10] and in agreement with CT studies [23], we found that patients with severe obesity had more than 1.2 cm pericardial fat around the right ventricular wall, with even higher amounts in diabetic patients. Despite a significant BMI reduction and an average 2.6 mm thinning of the pericardial fat layer 1 year after bariatric surgery, the amount of pericardial fat after surgery remained larger than what has previously been reported at lower BMIs [13], in particular in men and patients with persistently abnormal BMI.

### 4.3. Aortic Wall Changes in Severe Obesity and after Bariatric Surgery

Non-coronary aortic wall calcifications are associated with increased coronary risk and mortality, especially when present in the thoracic aorta [4]. Higher BMI has earlier been associated with subclinical atherosclerosis in the descending aorta, the carotid arteries, and the coronary tree of postmenopausal women [31]. In an experimental setting, obesity has recently been identified as a cause of increased AWT in Wistar rats [7]. Our study complements these previous reports by showing that the aortic root wall is significantly thicker in severely obese men and correlates with the pericardial fat thickness. Large weight loss through surgery is followed by a positive remodeling of the aortic root wall but is less effective in older patients and in those with 1-year BMI > 28 kg/m^2^.

### 4.4. Clinical Implications

In the context of high obesity prevalence worldwide with additional worsening during the COVID-19 pandemic, studying the impact of effective weight-reducing strategies as surgery on atherosclerosis progression is of increasing clinical and epidemiological importance. Our study demonstrates that severely obese patients without clinical CAD have a 2-fold increase in valve sclerosis compared to the general population, a high amount of pericardial fat, and increased AWT. All these markers of atherosclerosis decline during the first year after bariatric surgery, but less in older patients, in men, and in patients with more severe preoperative atherosclerosis or who did not achieve a BMI < 28 kg/m^2^ postoperatively. Our findings will contribute to a more refined follow-up of severely obese patients at higher atherosclerotic risk before and after weight-reducing interventions.

### 4.5. Study Limitations

CT has higher spatial resolution and the possibility to quantify the amount of coronary artery calcium and valve calcium as well as the total pericardial fat volume. This is an advantage also due to the uneven distribution of pericardial fat, with higher fat amounts in the atrioventricular and interventricular grooves. However, CT is restricted by less availability, higher cost, and exposure to radiation, and has limitations related to the maximum body size, making it less feasible in studying severely obese individuals. Transthoracic echocardiography provides the possibility to assess four prognostic markers of subclinical atherosclerosis in combination in order to estimate the extent of subclinical disease. Moreover, assessment of valve motion and thickening beyond calcification is more accurate by echocardiography than by CT due to higher temporal resolution.

## 5. Conclusions

Echocardiographic markers of subclinical atherosclerosis are highly prevalent in severely obese subjects and decrease 1 year after bariatric surgery. The most pronounced reverse remodeling is achieved by younger patients that reach a 1-year BMI < 28 kg/m^2^. Women attain a greater reduction in the amount of pericardial fat than men, independent of traditional CV risk factors as well as their pre- and postoperative BMI.

## Figures and Tables

**Table 1:** Clinical, biochemical and echocardiographic characteristics preoperatively and 1 year after bariatric surgery. Results are presented as mean ± standard deviation or percentages.

	Preoperatively	1-Year Postoperatively	*p*-Value
**Clinical data**			
Weight (kg)	121 ± 20	84 ± 16	<0.001
BMI (kg/m^2^)	41.8 ± 4.9	28.9 ± 4.7	<0.001
Heart rate (bpm)	74 ±13	65 ± 9	<0.001
Systolic BP (mmHg)	134 ± 10	126 ± 9	<0.001
Diastolic BP (mmHg)	87 ± 7	83 ± 5	<0.001
Preoperative hypertension	55%		na
Preoperative diabetes	16%		na
**% on medication**			
-Aspirin	3%	2%	0.03
-Lipid lowering treatment	14%	5%	<0.001
-Antihypertensive treatment	26%	15%	<0.001
-Antidiabetic treatment	15%	7%	<0.001
**Biochemical data**			
Serum creatinine (µmol/L)	71 ± 13	64 ± 11	<0.001
Serum total cholesterol (mmol/L)	4.7 ± 1.0	4.4 ± 0.9	0.06
Serum LDL cholesterol (mmol/L)	3.1 ± 0.9	2.8 ± 0.8	0.003
Serum triglycerides (mmol/L)	1.6 ± 0.7	1.0 ± 0.4	<0.001
Serum 25-hydroxyvitamin D	57.7 ± 24.0	68.9 ± 25.2	0.002
HbA_1c_ (%)	5.8 ± 1.2	5.4 ± 0.7	<0.001
**Echocardiographic data**			
LV end-diastolic diameter (cm)	5.06 ± 0.42	4.93 ± 0.42	<0.002
LV end-systolic diameter (cm)	3.41 ± 0.38	3.32 ± 0.35	0.06
LV mass index (g/m^2.7^)	44 ± 12	39 ± 11	<0.001
LV hypertrophy	35%	20%	<0.001
LV EF (%)	61 ± 5	59 ± 6	0.07
LV GLS (%)	−15.7 ± 4.7	−20.3 ± 2.7	<0.001
Pericardial fat (mm)	12.5 ± 3.7	9.9 ± 2.9	<0.001
Aortic root diameter (cm)	2.73	2.72	0.87
AWT (mm)	4.86 ± 1.74	4.37 ± 1.59	0.02

BMI, body mass index. BP, blood pressure. EF, ejection fraction. GLS, global longitudinal strain. HbA_1c_, glycated haemoglobin. LV, left ventricle. NA, not applicable.

**Table 2:** Predictors of reduction in pericardial fat and AWT 1 year after bariatric surgery identified in multivariate linear regression analysis run with backward stepwise removal of non-significant variables. Excluded variables in both models: age; preoperative diabetes, systolic and diastolic BP, BMI and AWT; 1-year systolic BP and 1-year reduction in heart rate.

1-Year Reduction in Pericardial Fat	R^2^ = 0.52, *p* < 0.001		
	**Beta**	***t***	***p*-Value**
Preoperative pericardial fat	0.71	8.88	<0.001
Female sex	0.16	2.04	0.04
1-year diastolic BP	−0.20	−2.34	0.02
1-year BMI	−0.16	−1.97	0.05
1-year reduction in LV mass	−0.14	−1.78	0.08
**1-Year Reduction in AWT**	**R^2^ = 0.50, *p* < 0.001**		
Age	−0.22	−2.82	<0.01
Preoperative AWT	0.71	8.87	<0.001
1-year BMI	−0.21	−2.58	0.01

**Table 3:** Predictors of persistent valve sclerosis (AVS and MVS) 1 year after bariatric surgery identified in binary logistic regression analyses run with backward stepwise removal of non-significant variables. Excluded variables in both models: age; sex; preoperative diabetes, systolic and diastolic BP, BMI and pericardial fat; 1-year BP and pericardial fat.

1-Year AVS	Nagelkerke R^2^ = 0.30, Wald 21, *p* < 0.001		
	**Wald**	***p*-Value**	**OR [95% CI]**
Age	6.56	0.01	1.08 [1.02–1.15]
1-year BMI	4.56	0.03	1.15 [1.01–1.30]
Preoperative AVS			
-mild	0.56	0.45	1.62 [0.46–5.69]
-moderate	4.44	0.04	6.31 [1.14–35.03]
**1-Year MVS**	**Nagelkerke R^2^ = 0.43, Wald 38, *p* < 0.001**		
1-year BMI	5.18	0.02	1.40 [1.05–1.87]
Preoperative systolic BP	3.26	0.07	0.89 [0.78–1.01]
Preoperative MVS	7.17	<0.01	35.57 [2.61–485.54]
Preoperative AVS	4.81	0.03	9.46 [1.27–70.53]

## Data Availability

The data underlying this article will be shared on reasonable request to the corresponding author.

## Abbreviations

AVS = aortic valve sclerosis; AWT = aortic wall thickness; BMI = body mass index; BP = blood pressure; CAD = coronary artery disease; CV = cardiovascular; EF = ejection fraction; FatWest = Bariatric Surgery on the West Coast of Norway; GLS = global longitudinal strain; LV = left ventricle; MVS = mitral valve sclerosis.
